# Polycystic lipomembranous osteodysplasia with sclerosing leukoencephalopathy: report of two cases

**DOI:** 10.1590/1980-5764-DN-2024-0198

**Published:** 2025-08-04

**Authors:** Joanisson Rubens Gomes Diniz, Katie Moraes de Almondes, Clécio Godeiro, Rodrigo Alencar e Silva

**Affiliations:** 1 Hospital Geral de Fortaleza, Fortaleza CE, Brazil.; 2 Universidade Federal do Rio Grande do Norte, Hospital Universitário Onofre Lopes, Aging Neuropsychology Service, Natal RN, Brazil.; 3 Universidade Federal do Rio Grande do Norte, Hospital Universitário Onofre Lopes, Departamento de Neurologia, Natal RN, Brazil.

**Keywords:** Dementia, Alzheimer Disease, Leukoencephalopathy, Case Reports, Demência, Doença de Alzheimer, Leucoencefalopatias, Relatos de Caso

## Abstract

The polycystic lipomembranous osteodysplasia with sclerosing leukoencephalopathy–Nasu-Hakola disease–is a hereditary and progressive pathology, which is mainly associated with pre-senile dementia and changes in bone architecture.

**Objective:**

To report two rare cases of sibling patients treated with early-onset dementia syndrome with genetic etiology, and to review the literature on the topic.

**Methods:**

Review of medical records, interviews and recording of the diagnostic methods to which patients were subjected. From this, a report was prepared of two cases who began behavioral changes in the third decade of life, who developed, at different times, symptoms of similar cognitive impairment. Bibliographic research carried out in the United States National Library of Medicine (PubMed), Medical Literature Analysis and Retrieval System Online (MEDLINE), Latin American and Caribbean Health Sciences Literature (LILACS), UpToDate and Scientific Electronic Library Online (SciELO) databases for bibliographic review.

**Results:**

Two clinical cases with genetic confirmation of Nasu-Hakola disease and a brief literature review were described.

**Conclusion:**

These cases illustrate a presentation of pre-senile dementia syndrome and reinforce the importance of adequate diagnosis for timely treatment, humanized multidisciplinary follow-up aiming to improve quality of life, as well as genetic and family counseling.

## RESUMO

A osteodisplasia lipomembranosa policística com leucoencefalopatia esclerosante – doença de Nasu-Hakola – é uma patologia hereditária, progressiva, que cursa com associação principal de demência pré-senil e alterações na arquitetura óssea.

**Objetivo:**

Relatar dois casos raros de pacientes irmãos atendidos com síndrome demencial de início precoce com etiologia genética e fazer revisão literária sobre o tema.

**Métodos:**

Revisão de prontuário, entrevistas, registro dos métodos diagnósticos aos quais os pacientes foram submetidos. Com base nisso, foi elaborado o relato de dois casos que iniciaram alteração comportamental na terceira década de vida, que evoluíram, em épocas distintas, com declínio cognitivo semelhante. Pesquisa bibliográfica realizada nas bases de dados Biblioteca Nacional de Medicina dos Estados Unidos (PubMed), Sistema Online de Busca e Análise de Literatura Médica (MEDLINE), Literatura Latino-Americana e do Caribe em Ciências da Saúde (LILACS), UpToDate e Scientific Electronic Library Online (SciELO) para revisão bibliográfica.

**Resultados:**

Foram descritos dois casos clínicos com confirmação genética da Doença de Nasu-Hakola e breve revisão da literatura.

**Conclusão:**

Estes casos ilustram uma apresentação de síndrome demencial pré-senil e reforçam a importância do diagnóstico adequado para tratamento oportuno e seguimento multidisciplinar humanizado, objetivando melhoria da qualidade de vida, assim como aconselhamento genético e familiar.

## INTRODUCTION

 Dementia syndromes are marked by a decline in memory and impairment in other cognitive domains potentially accompanied by behavioral changes. This decline must signify a departure from the individual’s previous pattern of functioning, negatively affect their social and/or occupational abilities^1^. 

 These syndromes have various subtypes and manifestations, with Alzheimer’s and vascular dementia being the most common. However, there are conditions that begin before the age of 65, known as pre-senile or early-onset dementia. The number of people diagnosed with pre-senile dementia has grown exponentially and, despite this increase, it remains poorly understood. Consequently, early-onset dementia is often underdiagnosed and inadequately treated^2^. 

 Leukodystrophies are a diverse group of genetically determined diseases that primarily affect the white matter. While they predominantly affect children and present in early childhood, these disorders can also occur in adults. Late-onset leukodystrophies may initially manifest with isolated psychiatric symptoms, behavioral changes, and cognitive deficits. Other clinical manifestations include motor signs, peripheral neuropathy, bulbar dysfunction, and epilepsy. Nasu-Hakola disease, also known as polycystic lipomembranous osteodysplasia with sclerosing leukoencephalopathy (PLOSL), is a rare, autosomal recessive adult leukodystrophy characterized by early-onset progressive dementia and recurrent bone fractures due to polycystic bone lesions. The disease typically begins in the third decade of life and leads to death within a few years^3^. 

 The objective was to report two rare cases of sibling patients treated for early-onset dementia syndrome with defined genetic etiology. 

## METHODS

 The case reports were prepared by reviewing medical records, interviewing patients and family members, and documenting diagnostic methods. 

 Bibliographic research of medical literature was conducted, covering case reports, review articles, original articles, and guidelines written in English and Portuguese. This research was sourced from the websites of the United States National Library of Medicine (PubMed), Medical Literature Analysis and Retrieval System Online (MEDLINE), Latin American and Caribbean Health Sciences Literature (LILACS), and Scientific Electronic Library Online (SciELO). 

 The descriptors used were: "dementia syndrome", "presenile dementia", "Nasu-Hakola disease", "polycystic lipomembranous osteodysplasia with sclerosing leukoencephalopathy". 

## CASE REPORTS

### Case 1

 A previously healthy 39-year-old man began experiencing behavioral changes at 28, including psychomotor agitation and disorientation. He started using illicit drugs like cocaine and crack and began getting lost frequently. Atypical antipsychotics were prescribed, and his condition was attributed to divorce stress. In terms of family history, there is a significant history of consanguinity in the family: both the parents and the paternal and maternal grandparents are first or second cousins. Furthermore, his paternal grandmother had Alzheimer’s disease (AD) with the first symptoms starting at 78 years of age. His parents are healthy. The patient is the oldest of four siblings: three males and one female. The other two male siblings are healthy and the youngest sister will be reported in case 2. 

 After six years, he developed tonic-clonic seizures and was prescribed valproic acid. He exhibited additional symptoms like tremors, rigidity, and bradykinesia, and continued using cocaine and marijuana. 

 In the following months, he experienced loss of functionality, needing assistance with dressing and bathing. Dysautonomic symptoms, including urinary incontinence, constipation, cold sweats, perseveration of ideas, disinhibition, and sexual compulsion, emerged. 

 He had difficulty articulating words, his thinking became disorganized, and he exhibited echolalia and perseveration of topics he heard on television. 

 After a year without seizures, they recurred and became more refractory. His cognition and communication worsened, making him dependent on others for all basic activities of daily living (ADLs). He began to lose weight due to dysphagia and could no longer walk. 

**Figure 1:**
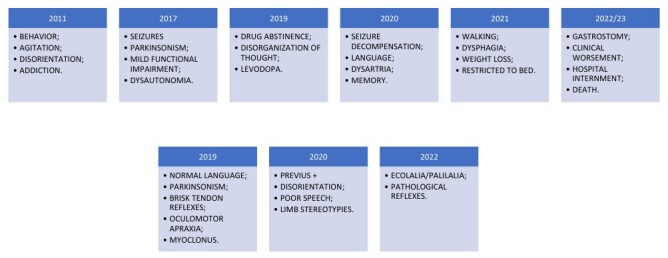
Summary of the patient’s clinical evolution. In the upper part there are signs and symptoms and, in the lower part, a physical neurological examination.

 The patient was admitted for gastrostomy surgery and developed a hospital-acquired infection, needing ventilatory support and a tracheostomy. After discharge, he returned home but died months later from respiratory infection complications (see Figure 1). 

 Diagnostic evaluation included general biochemistry, serology, iron and calcium kinetics, serum and urinary copper levels, ceruloplasmin, thyroid function tests, vitamin D and B12 levels, cerebrospinal fluid (CSF) analysis, brain magnetic resonance imaging (MRI), electroencephalogram (EEG), x-rays of hands, wrists, feet, and ankles, bone densitometry, and whole exome sequencing. 

**Figure 2:**
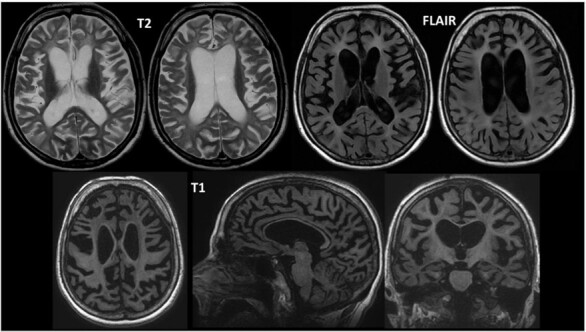
There is brain volumetric reduction predominantly in the supratentorial compartment, associated with compensatory dilation of the lateral and third ventricles. Diffuse leukoencephalopathy suggestive of hypomyelination is present, along with marked atrophy of the basal ganglia and increased iron accumulation in the globus pallidus. There are signs of diffuse atrophy in the cerebral cortex and a generalized reduction in the thickness of the corpus callosum, especially in the body and genu. The overall appearance is compatible with supratentorial leukoencephalopathy with hypomyelination and basal ganglia atrophy.

 Laboratory tests showed no significant changes. The brain MRI showed diffuse atrophy and signs of leukoencephalopathy (Figure 2). The EEG revealed moderate, diffuse, and focal slowing, predominantly in the posterior regions, accompanied by frequent epileptiform abnormalities in the bilateral parieto-occipital regions, with a predominance on the right side. Whole exome sequencing identified a homozygous pathogenic variant NM 018965.3: c.154C>T; p.(Arg52Cys) in the *TREM2* gene (*triggering receptor expressed in myeloid cells 2*), which is inherited in an autosomal recessive manner. The variant, located at chromosomal position chr6: 41.129.238, is clinically consistent with PLOSL. 

### Case 2

 Woman, 34 years old, single, ten years of schooling, no previous work activity. At the beginning of the third decade of life, she began psychomotor agitation with aggression associated with disorganized thinking (unable to maintain a dialogue, inserting subjects into the conversation outside a given context), wandering behavior (entering and leaving the same room or moving between areas at home repeatedly) and disorientation in time/space. 

 Her condition progressed over the years, resulting in inattention, disinhibition, hypersexuality, loss of insight, and infantilization. Additionally, she developed depressive symptoms, including emotional lability, irritability, and social isolation. These issues emerged in a context where the family was already overwhelmed by caring for a brother with dementia. During a psychiatric evaluation, olanzapine and bupropion were prescribed, but there was no improvement in her behavior. 

 She developed amnesia, urge urinary incontinence, language changes, apathy and executive dysfunction, leading to functional decline and dependence on family members for assistance with ADLs. There have been no changes in sleep, gait, falls, or hallucinations since the onset of his condition. 

**Figure 3:**
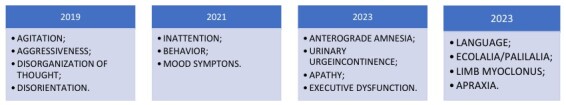
Summary of the patient’s clinical evolution. In the upper part there are signs and symptoms and, in the lower part, a physical neurological examination.

 On examination, she exhibited echolalia and palilalia, along with myoclonus in the upper limbs. On the Mini-Mental State Examination (MMSE), she scored 10 out of 30 points, losing 9 points in orientation, 5 in attention and calculation, 3 in recall memory, and 5 in language. She has a history of distal edema in the lower limbs and a right ankle fracture from a fall at ground level (see Figure 3). 

 The clinical examination performed in this case was identical to that in case 1. Laboratory tests did not reveal any significant changes. Bone densitometry, CSF analysis, and radiographs of the hands, wrists, feet, and ankles were all normal. 

**Figure 4:**
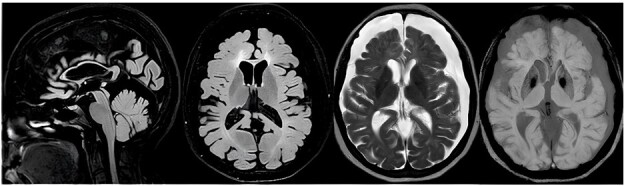
Chronic subdural collections, located in the cerebral convexities and on the cerebellar hemispheres; brain volumetric reduction associated with ectasia of the supratentorial ventricular system; supratentorial leukoencephalopathy with hypomyelination and significant thinning of the corpus callosum, with the exception of the splenium.

 Brain MRI showed atrophy and supratentorial leukoencephalopathy (Figure 4). The EEG showed no changes and the whole exome sequencing revealed a pathogenic variant identical to that seen in the examination of case 1. Nasu-Hakola disease (NHD), a rare form of adult leukodystrophy also known as polycystic lipomembranous osteodysplasia with sclerosing leukoencephalopathy, is an inherited systemic disease characterized by multiple cyst-like bone lesions and progressive, severe leukoencephalopathy that results in significant neurological impairment. The first patients and subsequent cases were initially described in Finland and Japan. However, the disease is now recognized globally, with additional occurrences reported in countries including Norway, the United States of America, Italy, and Brazil^4^. 

 The major causes of young-onset dementia are AD, vascular dementia and frontotemporal lobar degeneration (FTLD)^5^. Most studies identify vascular dementia^6^ as the most common etiology for the presented conditions. However, considering the literature that describes behavioral onset, vascular dementia is unlikely to be the primary diagnosis. Instead, FTLD is a neuroanatomically and pathologically heterogeneous group of neurodegenerative diseases that initially and preferentially affect the frontal and temporal lobes of the brain, and it has clinical characteristics that resemble the presented cases, mainly the behavioral variant of frontotemporal dementia (bvFTD), characterized by neuropsychiatric symptoms^5,7^. On the other hand, the history of consanguinity and the brain MRI findings, such as signs of leukoencephalopathy and thinning of the corpus callosum, led us to consider another diagnostic hypothesis. 

 The NHD is thought to arise from dysfunction in microglia, crucial immune and inflammatory agents acting as brain macrophages within the central nervous system (CNS). Dysfunctional microglia are implicated in the onset of various neurological and psychiatric disorders. Recently, NHD has been linked to mutations in the *DAP12*DAP12 gene (also known as *TYROBP*) or the *TREM2* gene, which encode proteins and receptors expressed in human microglia. These genes also influence the correct differentiation and function of osteoclasts, which accounts for the bone manifestations observed^8^. 

 NHD was initially described separately by Nasu and Hakola around the 1970s. To date, around two hundred cases have been diagnosed worldwide^9^. 

 From the age of 20, initial symptoms typically include pain and swelling in the lower limbs, stemming from alterations in bone architecture in the wrists and ankles. These changes can increase susceptibility to bone fractures in the extremities following minor impacts. Around the age of 30, patients begin to experience a gradual onset of neuropsychiatric symptoms, which may encompass epileptic seizures, agnosia, apraxia, speech disorders, memory impairment, euphoria, and diminished social inhibitions^10^. 

 The disease progresses through four stages: latent, bone, early neurological, and late neurological. After normal childhood development (latent stage), symptoms usually begin in adolescence or early adulthood with polyarthralgias. This phase is often mistaken for other common bone pathologies due to the absence of significant neuropsychiatric changes at this stage. During the bone stage, typically occurring between 15 and 30 years of age, patients experience pain in the hands, wrists, ankles, and feet, with bone fractures often occurring due to minor trauma^9^. Classic bone lesions are not always detectable on radiography, as observed in the two cases presented in this article. Currently, there are no studies indicating the prevalence of patients with NHD who do not exhibit classic bone lesions^11^. 

 During the early neurological stage, typically in the third or fourth decade of life, patients develop personality changes characteristic of frontotemporal dementia (FTD). Initially subtle and noticeable only to close associates, these behavioral changes gradually become more pronounced over months to years^12^. Patients show infantilized and playful behavior, lack insight, and exhibit social inhibition and impulsive behaviors. They commonly experience marked inattention and memory deficits. Language changes include increased speech speed and various types of aphasia, often advancing to global aphasia. As the disease progresses, symptoms of higher cortical dysfunction, such as memory impairments, sensory agnosias, agraphia, acalculia, and apraxia. Other neurological changes include tremors, upper motor neuron abnormalities, gait disturbances, re-emergence of primitive reflexes, peripheral neuropathy, generalized tonic-clonic seizures, and myoclonus^4^. 

 In the late neurological stage, patients progress to severe dementia. They become bedridden, unable to walk due to bone pain and the severity of neurological changes. NHD leads to progressive dementia that is usually fatal during the fifth decade of life due to respiratory or urinary complications^9^. 

 Laboratory and CSF analyses typically show no abnormalities in NHD. There are no reported characteristic abnormalities in hematological and urine biochemical analyses, or in liver and kidney functions. Levels of pituitary, thyroid, parathyroid, adrenal, and sex hormones, as well as serum cholesterol, triglycerides, phospholipids, free fatty acids, and lipoproteins, generally remain within normal limits^4^. 

 The bone changes typically observed in most patients with NHD are identified through x-rays. These may reveal multiple cystic bone lesions, predominantly located at the ends of the bones in the upper and lower limbs. The lesions initially involve the reduction of bone trabeculae in the epiphysis and metaphysis, leading to detectable bone rarefactions on examination. Subsequently, these lesions progress to form cystic areas with poorly defined margins and without sclerotic borders^4^. 

 Neuroimaging tests usually detect changes only months or years after the onset of the initial neurological stage. Brain computed tomography shows progressive cortical atrophy, especially in the frontal areas and the polar region of the temporal lobes, along with abnormal calcifications in the basal ganglia^12,13^. T1-weighted MRI images show cerebral atrophy, primarily affecting the frontal areas, along with thinning of the corpus callosum and ventricular enlargement unrelated to atrophy. T2-weighted images additionally demonstrate nonspecific, diffuse signal changes in the white matter and signal reduction in the basal ganglia, potentially linked to the observed calcium and iron deposits in these regions. FLAIR and spin echo sequences reveal a diffuse, nonspecific increase in white matter signal, characteristic of the disease’s leukodystrophy^14^. 

*TREM2*, initially identified as a receptor associated with *DAP12* on macrophages and dendritic cells, is also expressed on osteoclasts and microglia, the central nervous system’s resident immune cells. Recent studies have linked *TREM2* to certain genetic forms of AD. However, NHD and AD are distinct, with NHD showing symptoms like early onset, painful bone cysts, limb fractures, and sclerosing leukoencephalopathy, which differ from AD. *TREM2* is involved in the phagocytic function of microglia against amyloid plaques, and reduced *TREM2* activity may impair the clearance of these toxic substances, leading to brain damage^15^. 

 Based on described cases, we emphasize that Nasu-Hakola disease, a rare cause of dementia in our country, should be considered in a pre-senile context. Its phenotypic presentation resembles FTLD, with leukoencephalopathy signs on neuroimaging and corpus callosum thinning, even if bone changes are initially absent on radiography. 

## Data Availability

Data sharing is not applicable.

## References

[1] Arvanitakis Z, Shah RC, Bennett DA (2019). Diagnosis and Management of Dementia: Review. JAMA.

[2] Lambert MA, Bickel H, Prince M, Fratiglioni L, Von Strauss E, Frydecka D (2014). Estimating the burden of early onset dementia; systematic review of disease prevalence. Eur J Neurol.

[3] Xing J, Titus AR, Humphrey MB (2015). The TREM2-DAP12 signaling pathway in Nasu-Hakola disease: a molecular genetics perspective. Res Rep Biochem.

[4] Bianchin MM, Capella HM, Chaves DL, Steindel M, Grisard EC, Ganev GG (2004). Nasu-Hakola disease (polycystic lipomembranous osteodysplasia with sclerosing leukoencephalopathy--PLOSL): a dementia associated with bone cystic lesions. From clinical to genetic and molecular aspects. Cell Mol Neurobiol.

[5] Ljubenkov PA, Miller BL (2016). A clinical guide to frontotemporaldementias. Focus (Am Psychiatr Publ).

[6] Rossor MN, Fox NC, Mummery CJ, Schott JM, Warren JD (2010). The diagnosis of young-onset dementia. Lancet Neurol.

[7] Lanata SC, Miller BL (2016). The behavioural variant frontotemporal dementia (bvFTD) syndrome in psychiatry. J Neurol Neurosurg Psychiatry.

[8] Ohgidani M, Kato TA, Setoyama D, Sagata N, Hashimoto R, Shigenobu K (2014). Direct induction of ramified microglia-like cells from human monocytes: dynamic microglial dysfunction in Nasu-Hakola disease. Sci Rep.

[9] Xing J, Titus AR, Humphrey MB (2015). The TREM2-DAP12 signaling pathway in Nasu-Hakola disease: a molecular genetics perspective. Res Rep Biochem.

[10] Kondo T, Takahashi K, Kohara N, Takahashi Y, Hayashi S, Takahashi H (2002). Heterogeneity of presenile dementia with bone cysts (Nasu-Hakola disease): three genetic forms. Neurology.

[11] Bock V, Botturi A, Gaviani P, Lamperti E, Maccagnano C, Piccio L (2013). Polycystic lipomembranous osteodysplasia with sclerosing leukoencephalopathy (PLOSL): a new report of an Italian woman and review of the literature. J Neurol Sci.

[12] Mäkelä P, Järví O, Hakola P, Virtama P (1982). Radiologic bone changes of polycystic lipomembranous osteodysplasia with sclerosing leukoencephalopathy. Skeletal Radiol.

[13] Iivanainen M, Hakola P, Erkinjuntti T, Sipponen JT, Ketonen L, Sulkava R (1984). Cerebral MR and CT imaging in polycystic lipomembranous osteodysplasia with sclerosing leukoencephalopathy. J Comput Assist Tomogr.

[14] Brenner C, Speck-Martins CE, Brum JM, Lucato LT, Leite C da C (2014). Computed tomography and magnetic resonance imaging in the osseous phase of Nasu-Hakola disease. Arq Neuropsiquiatr.

[15] Jonsson T, Stefansson H, Steinberg S, Jonsdottir I, Jonsson PV, Snaedal J (2013). Variant of TREM2 associated with the risk of Alzheimer’s disease. N Engl J Med.

